# The longitudinal impact of emotional intelligence and psychological empowerment on work engagement among university administrators: a cross-lagged panel model approach

**DOI:** 10.3389/fpsyg.2025.1667110

**Published:** 2026-01-12

**Authors:** Lanfeng Zhou, Xinyu Wang

**Affiliations:** 1 College of Marxism, Putian University, Putian, Fujian, China; 2 School of Education and Psychology, Minnan Normal University, Zhangzhou, Fujian, China

**Keywords:** cross-lagged panel model, emotional intelligence, gender difference, psychological empowerment, work engagement

## Abstract

**Background:**

University administrators face numerous work-related challenges that can impact their emotional intelligence, psychological empowerment, and work engagement. While research on these factors is extensive, longitudinal studies investigating their reciprocal relationships remain limited, and even fewer have examined whether these dynamics differ by gender. This study addresses these gaps by exploring how emotional intelligence, psychological empowerment, and work engagement evolve among male and female university administrators in Putian City, China.

**Methods:**

A two-wave longitudinal design was used, with data collected at the start (August 2024 – T1) and end (February 2025 – T2) of an academic semester. This resulted in an overlapping sample of 416 participants who completed surveys at both time points. Validated scales were employed to measure emotional intelligence, psychological empowerment, and work engagement. Cross-lagged panel model (CLPM) and multiple-group analysis were performed to assess the variables’ longitudinal relationships and test for gender differences.

**Results:**

The findings revealed significant cross-lagged effects: emotional intelligence and psychological empowerment at T1 positively predicted work engagement at T2. Psychological empowerment showed the strongest contribution to later work engagement. Multi-group analyses indicated that these longitudinal associations were highly similar for male and female administrators, with no statistically significant gender differences in path strengths.

**Conclusion:**

The findings highlight the dynamic interplay between emotional intelligence, psychological empowerment, and work engagement over time, with both emotional intelligence and psychological empowerment contributing to increased engagement among university administrators. The longitudinal associations were broadly similar for male and female administrators, suggesting that interventions aimed at strengthening emotional and psychological resources may benefit the administrative workforce as a whole.

## Introduction

Work engagement is a critical psychological state that promotes job performance, employee retention, and organizational success (Schaufeli et al., 2002; Corbeanu and Iliescu, 2023; Memon et al., 2016). Defined by vigor, dedication, and absorption, work engagement has been widely studied in faculty and corporate populations (Schaufeli et al., 2002; Shimazu et al., 2012; González-Rico et al., 2022; Gürbüz et al., 2023). Still, it remains underexplored among university administrators, a group that plays a pivotal yet often invisible role in institutional functioning. These professionals are responsible for mediating between academic leadership and operational execution, managing diverse stakeholders (faculty, students, external agencies), and navigating complex bureaucratic systems (Thang and Tuyen, 2023; Asiri et al., 2012). As such, they operate under persistent time pressure, emotional labor, and role conflict, conditions that directly threaten sustained engagement and can manifest as psychological challenges such as stress, burnout, and role ambiguity (Peretomode, 2012; Gillespie et al., 2001). Within the Job Demands–Resources (JD-R) framework (Bakker and Demerouti, 2007), these challenges are conceptualized as job demands, whereas personal and job resources can buffer their effects. To more clearly align this occupational profile with the JD-R model, university administrative work inherently involves high job demands, including bureaucratic complexity, heavy service loads, constant problem-solving across multiple stakeholders, and frequent emotional regulation in difficult interactions. At the same time, their access to job resources, including autonomy, role clarity, institutional recognition, and relational support, is often limited compared with that of academic staff (Wray and Kinman, 2022; González-Rico et al., 2022). Despite their critical institutional functions, university administrators have received far less scholarly attention than faculty members, largely because research in higher education traditionally prioritizes teaching and research outcomes over administrative well-being. Empirical evidence has demonstrated that administrative personnel in universities experience heavier bureaucratic workloads, greater emotional labor, and stricter hierarchical pressure than academic staff (Lei et al., 2023; Ahmad et al., 2025; Pathiranage et al., 2024; Li and Ye, 2021). However, these findings remain fragmented and primarily descriptive, suggesting a lack of theoretically grounded models that explain how administrators maintain engagement in these demanding contexts. By integrating JD-R, emotional intelligence theory (Bakker and Demerouti, 2007), psychological empowerment theory (Spreitzer, 1995), and gender role theory (Eagly, 2013), this study advances existing knowledge by proposing a framework that simultaneously considers personal, job, and cultural role expectations as drivers of work engagement.

Within this context, emotional intelligence, the ability to perceive, understand, and regulate emotions, has emerged as a vital personal resource that can protect against emotional exhaustion and promote adaptive coping (Zeidner et al., 2004). Similarly, psychological empowerment, the intrinsic experience of competence, autonomy, meaning, and impact, is a critical job resource that fuels intrinsic motivation and fosters commitment (Menon, 1999). However, current research has two major limitations. First, most studies linking emotional intelligence and psychological empowerment to work engagement are cross-sectional, precluding insight into temporal dynamics or the directionality of relationships. Second, reciprocal pathways among these constructs remain under-theorized, despite growing evidence that psychological states co-evolve and reinforce one another in the workplace (Xanthopoulou et al., 2009). Despite growing research on emotional intelligence and psychological empowerment, university administrators remain understudied, particularly in Chinese higher education contexts. These administrators face complex operational and relational demands (Branson et al., 2016; Conway, 2012), yet the dynamic interplay between personal and job resources in sustaining their engagement over time remains poorly understood. To address this gap, the present study employs a two-wave longitudinal design to examine reciprocal relationships between emotional intelligence, psychological empowerment, and work engagement among university administrators in Putian City, China. The study further investigates gender as a potential moderator and applies structural equation modeling and multiple-group analysis, providing culturally grounded evidence from an underrepresented population and advancing both theoretical understanding and practical strategies to enhance administrator engagement.

## Theoretical framework and literature review

### JD-R model as the overarching framework

The JD-R model provides a comprehensive foundation for examining how work-related resources and demands shape employee outcomes. JD-R theory posits that every job has specific risk factors associated with job stress, categorized broadly as demands and resources (Bakker and Demerouti, 2007; Schaufeli and Bakker, 2004). To better reflect the realities of higher education administration, this study explicitly conceptualizes job demands and resources within the administrative context. University administrators frequently face bureaucratic complexity, including navigating multi-layered approval procedures; conflicting stakeholder expectations from faculty, students, and institutional leaders; constant pressure to deliver services; and substantial emotional labor arising from frontline conflict management (Dourgkounas, 2025; Lei et al., 2023; Gauntner, 2013; Veles et al., 2023; Şahin Özan and Akin, 2024). These demands represent a high cognitive and emotional load, consistent with JD-R’s definition of energy-depleting requirements. Conversely, administrators’ job resources often include relational support from supervisors and colleagues, institutional recognition, access to decision-making authority, and opportunities for competency development (Schaufeli and Taris, 2013; Owens, 2022; Smagina and Stich, 2025). However, in many Chinese universities, these resources are unevenly distributed due to hierarchical structures and limited autonomy. Psychological empowerment captures these job resources by reflecting internalized perceptions of meaning, competence, self-determination, and impact, all of which are essential for offsetting administrative demands (Spreitzer, 1995). Emotional intelligence, as a personal resource, further enables administrators to regulate emotions, manage interpersonal complexities, and sustain motivation despite competing demands (Goleman, 1996). Emotional intelligence is conceptualized as a personal resource that enables emotional regulation and interpersonal competence (Joseph and Newman, 2010), whereas psychological empowerment is a job resource reflecting meaning, autonomy, and control (Tuckey et al., 2012). Both are hypothesized to contribute individually, interactively, and developmentally to work engagement.

### Emotional intelligence and work engagement

Emotional Intelligence Theory asserts that emotional intelligence comprises self-awareness, self-regulation, social awareness, and relationship management (Goleman, 1996). In emotionally demanding roles such as university administration, emotional intelligence enables individuals to manage stress, defuse conflict, and regulate interpersonal dynamics, all of which are essential to sustained engagement (Beytekin, 2021; Sharma and Sehrawat, 2014). Several empirical studies have confirmed that emotional intelligence positively predicts work engagement. For instance, Brunetto et al. (2012) found that higher emotional intelligence among public sector employees was associated with greater engagement, particularly under emotionally charged conditions. Similarly, Lu et al. (2021) found that emotional intelligence buffered the negative impact of emotional labor among Korean education administrators. Meta-analyses have also concluded that emotional intelligence shows a medium-to-strong positive correlation with work engagement across occupational groups (Miao et al., 2017). In Chinese culture, emotional restraint is culturally encouraged, and indirect communication is the norm (Nisbett et al., 2012)(Eckhardt, 2002). Thus, emotionally intelligent administrators are better equipped to navigate institutional hierarchies and relational subtleties, thereby sustaining engagement.

*H1: Emotional intelligence at Time 1 will significantly and positively predict work engagement at Time 2 among university administrators.*

### Psychological empowerment and work engagement

Psychological Empowerment Theory defines psychological empowerment as a motivational state comprising four dimensions: meaning, competence, self-determination, and impact (Spreitzer, 1995). When employees perceive their work as purposeful, feel capable of performing it, and believe they have autonomy and influence, they are more likely to remain committed and energized (Salah ud din Khan et al., 2025). Psychological empowerment has been consistently associated with work engagement in education and public administration. For instance, Meng and Sun (2019) found that higher empowerment correlated with higher engagement among university faculty, primarily through meaning and competence. Likewise, Laschinger and Finegan (2005) demonstrated this relationship in health administration. Empowerment is especially impactful in hierarchical organizational settings, where autonomy and self-efficacy are not automatically granted but must be developed over time, making them particularly influential in sustaining engagement. Moreover, psychological empowerment may be especially relevant for university administrators in non-Western hierarchical cultures such as China, where formal authority is concentrated, and feelings of autonomy and competence are hard-earned and highly valued (Sharma and Sehrawat, 2014) (Li et al., 2010). Consequently, the current study hypothesizes that:

*H2: Psychological empowerment at Time 1 will significantly and positively predict work engagement at Time 2 among university administrators.*

### Reciprocal relationship between emotional intelligence and psychological empowerment

While emotional intelligence and psychological empowerment have primarily been studied as separate predictors of work engagement, emerging theory and evidence suggest that these constructs may reinforce each other over time. From a cognitive–motivational–relational perspective, individuals with high emotional intelligence are better able to regulate stress and manage social interactions. This may increase feelings of competence and impact, two core components of empowerment (Gong et al., 2020). Conversely, when employees feel psychologically empowered, they experience greater control and purpose in their roles, which enhances confidence and emotional stability, components of trait emotional intelligence (Hameli et al., 2023). Empirical studies support this bidirectional association. For instance, Alotaibi et al. (2020) found that empowerment interventions improved self-efficacy and emotional regulation capacity. Likewise, Salovey and Grewal (2005) argued that emotional intelligence skills are reinforced through positive work experiences, including autonomy and meaning, both of which are central to psychological empowerment. In administrative roles that involve relational and cognitive demands, it is plausible that emotional intelligence and psychological empowerment mutually enhance each other over time.

*H3: Emotional intelligence and psychological empowerment will exhibit reciprocal, positive relationships over time.*

### Gender as a moderator

Gender is an important but underexamined factor in understanding how emotional intelligence and psychological empowerment contribute to work engagement. Gender Role Theory highlights that organizational expectations and socialization patterns shape how men and women navigate emotional expression, autonomy, and interpersonal demands at work (Stockard, 2006; Nixon et al., 2011). In Chinese higher education administration, gendered norms and structural constraints, such as unequal access to decision-making roles and differentiated performance expectations, may influence how individuals experience emotional and psychological resources (Meng and Sun, 2019; Taylor et al., 2022). Thus, emotional intelligence and psychological empowerment may not operate uniformly across genders. Examining both male and female administrators enables a more balanced understanding of whether these resources contribute similarly to engagement within this cultural and institutional context. This approach allows the study to test gender differences empirically without relying on assumptions and ensures that findings reflect actual patterns within Chinese university administration. Therefore, the present study hypothesizes:

*H4*: Gender will moderate the reciprocal relationships among emotional intelligence, psychological empowerment, and work engagement among Chinese university administrators.

### This study

Despite the proliferation of research on emotional intelligence, psychological empowerment, and work engagement, several critical gaps remain unaddressed, particularly in university administration. This role is cognitively complex, emotionally demanding, and culturally situated (Bachman and Grady, 2016; Lei et al., 2023; Vere et al., 2024). This study seeks to fill these gaps through a theoretically integrated, methodologically robust, and contextually grounded investigation. First, most existing studies examining the links between emotional intelligence, psychological empowerment, and work engagement are cross-sectional, limiting causal inference and obscuring the dynamic nature of psychological processes at work. As psychological states such as empowerment and engagement fluctuate over time in response to shifting interpersonal and institutional conditions, there is a pressing need for longitudinal studies examining how these constructs change and how they influence one another. This study uses a two-wave cross-lagged panel design to uncover directionality and mutual influence, extending the literature beyond static associations. Second, research on work engagement has focused primarily on faculty, teachers, and corporate employees, with little attention to university administrators. This population faces distinct challenges related to emotional labor, institutional hierarchy, and conflicting role demands. University administrators are central to the functioning of higher education institutions, yet their psychological health and motivational dynamics remain understudied. Importantly, similar patterns are also observed across sectors, where research has traditionally concentrated on “core” professional roles, such as clinicians in healthcare or managers in corporate settings, while allocating far less empirical attention to support or administrative personnel. Evidence from public administration and organizational psychology shows that support staff often experience greater emotional labor, reduced autonomy, and weaker professional identity than core staff, placing them at heightened risk of diminished engagement. Positioning university administrators within this broader category of understudied support employees enhances the practical relevance of this work and demonstrates how insights from higher education can contribute to wider cross-sector discussions on employee engagement. This research focuses specifically on administrators in Chinese universities, offering insights into a neglected but pivotal occupational group. Third, while emotional intelligence and psychological empowerment have each been linked to work engagement, their interrelationship has rarely been conceptualized as a reciprocal one. Most studies assume unidirectional influence (e.g., Emotional intelligence → Psychological empowerment), overlooking theoretical frameworks (e.g., affective events theory, cognitive-behavioral models) that support mutual reinforcement. This study makes a novel theoretical contribution by modeling bidirectional paths between emotional intelligence and psychological empowerment, suggesting that emotional competencies and empowerment perceptions may co-evolve over time. Lastly, although gender differences in emotional labor, access to empowerment, and affective regulation are well documented across occupational sectors, few studies have examined whether gender moderates the relationships among emotional intelligence, psychological empowerment, and work engagement. By applying Gender Role Theory to both male and female administrators, this study adopts a balanced, gender-inclusive approach that avoids privileging a single gender and ensures a scientifically neutral examination of potential moderating effects.

## Methods

### Study design

This study employed a two-wave longitudinal design to investigate the dynamic interplay between emotional intelligence, psychological empowerment, and work engagement among university administrators across Putian City, Fujian Province, China. A two-wave design was selected because it provides the minimum temporal structure necessary to test directional relationships in cross-lagged panel models while reducing participant burden and attrition risk, particularly important in administrative populations with heavy workloads. Data were collected in August 2024 (T1) and in February 2025 (T2), corresponding to the start and end of an academic semester, respectively. A six-month interval was selected because prior longitudinal studies on emotional intelligence, psychological empowerment, and work engagement have shown that meaningful changes in these psychological resources typically occur within medium-term periods of 4–8 months (Kim et al., 2018; Extremera et al., 2018). In university administrative settings, workload cycles, policy implementation periods, and staffing changes also follow semester-based timelines, making 6 months an appropriate interval for detecting fluctuations in work-related psychological states. Although longer time frames may reveal stronger causal patterns, the 6-month interval used here aligns with recent longitudinal research examining dynamic workplace processes.

### Population and eligibility criteria

The target population consisted of full-time professional staff in university administration, including roles in student affairs, academic affairs, institutional operations, and administrative coordination. Job titles varied across institutions and included mid-level administrative managers and officers responsible for operational and support functions. Senior academic leadership positions (e.g., provosts, deans) were excluded. Eligible participants: Were aged 18 or older, held administrative roles for at least 1 year, and were not on leave during the semester under study. Those in purely academic (teaching/research) or temporary positions were excluded.

### Sampling strategy and representativeness

A cluster sampling approach was used, with universities in Putian City serving as naturally occurring clusters. A purposive selection of 14 universities (nine public, five private) was made based on access and administrative cooperation. These institutions vary in size, governance structures, and the distribution of administrative roles, offering a reasonable representation of the broader administrative workforce across medium-sized Chinese cities. All full-time administrators were invited to participate in each selected university. This approach allowed for broad coverage across institutional types while accounting for operational feasibility. The clustered nature of the data was addressed analytically using design-based corrections in structural equation modeling.

### Response and retention

Eight hundred twenty-one individuals completed the T1 survey, and 609 completed the T2 survey. The final matched sample comprised 416 participants who completed both waves (50.7% retention). After excluding responses with >10% missing data or patterned responses, the final analytic sample (*N* = 416) had a gender composition of 52.5% female and a mean age of 31.56 (SD = 6.9).

### Missing data handling

Missing data were handled using full information maximum likelihood (FIML) in Mplus, which provides unbiased parameter estimates under missing-at-random assumptions and is recommended for longitudinal SEM. This approach ensured that all available information contributed to the analyses.

### Attrition bias assessment

To assess potential attrition bias, we conducted independent samples *t*-tests and chi-square tests comparing those who participated at both waves (*n* = 416) versus dropouts (*n* = 405). The results indicated no significant differences between the groups (*p* > 0.05), suggesting that attrition did not introduce systematic bias into the study. Therefore, the remaining sample represents the initial cohort, and the findings are generalizable within the study context.

### Power and sample adequacy

*A priori* power analysis using Monte Carlo simulation in Mplus (with *α* = 0.05, power = 0.80, expected path coefficient = 0.20) suggested a minimum sample size of 350 for detecting medium effects in cross-lagged panel models (Wolf et al., 2013; Muthén and Muthén, 2002). For gender moderation, multigroup SEM power estimates recommend at least 150 cases per group (Meade and Bauer, 2007; Kline, 2023), which was met in both the male (*n* ≈ 198) and female (*n* ≈ 218) subgroups. Thus, the final sample size (*N* = 416) was deemed adequate for both primary and subgroup analyses.

### Measuring instruments

#### Psychological empowerment

The Psychological Empowerment Scale (PES) by Spreitzer (1995), consisting of 12 items, is a widely used instrument in organizational psychology to assess employees’ feelings of empowerment in their work roles, including dimensions of meaning, competence, self-determination, and impact. Respondents rate each item on a 7-point Likert scale from “Strongly Disagree” to “Strongly Agree,” with higher scores reflecting greater perceived empowerment. Although originally developed in a Western context, a Chinese version of the PES has been adapted and validated among Chinese employees, including higher-education personnel, demonstrating acceptable factorial validity and reliability (Li-Chaoping and Shi-Kan, 2006; Meng and Sun, 2019). Therefore, the PES is deemed culturally appropriate and psychometrically suitable for assessing psychological empowerment among Chinese university administrators in the present study.

#### Emotional intelligence

A self-reported emotional intelligence scale (EIS) developed by Schutte et al. (1998), validated by Shi and Wang (2007), was adopted to assess the participants’ emotional intelligence. The EIS comprised 33 items, with three items scored in reverse. Participants were required to respond to the extent of their agreement or disagreement with each item on a 5-point Likert scale (strongly disagree to strongly agree). The final score is obtained by summing the responses to the items, with higher values indicating higher emotional intelligence. Several empirical investigations have demonstrated good validity and reliability across different settings (Birks et al., 2009; Zaman et al., 2021; Nightingale et al., 2018; Akhtar et al., 2023; Imran et al., 2013).

#### Work engagement

The Utrecht Work Engagement Scale (UWES) developed by Schaufeli et al. (2003) is a well-established tool used to measure work engagement, which refers to an individual’s level of enthusiasm, dedication, and absorption in their work. For the current study, we employed a short Chinese version of UWES (9 items) consisting of three key dimensions (Fong and Ng, 2012): Vigor, Dedication, & Absorption as initially designed. Participants’ response is recorded on a 7-point Likert scale from “Never” to “Always,” higher scores indicate higher levels of work engagement in the respective dimensions. The UWES has demonstrated high reliability and validity in various studies of organizational research and employee well-being (Schaufeli and Bakker, 2004; Fong and Ng, 2012; Mazzetti et al., 2023; Xanthopoulou et al., 2007), making it a robust measure of work engagement.

### Ethical protocols

This study was conducted in accordance with the ethical principles of the Declaration of Helsinki. Prior to data collection, ethical approval was obtained from the Research Ethics Committee of Putian University (No. 5798/R). Informed consent was obtained electronically. Participants were informed about the voluntary nature of their involvement, assured of their anonymity and confidentiality, and given the right to withdraw from the study at any point without penalty.

### Analytical approach

Cross-lagged path models, accounting for longitudinal effects while controlling for other variables, were employed to investigate the relationships among emotional intelligence, psychological empowerment, and work engagement. As both emotional intelligence and psychological empowerment are multidimensional constructs, we created parcels by averaging relevant items within each dimension to simplify the analysis while maintaining the integrity of the constructs. This approach allows for a more manageable model while still capturing the essential structure of the variables. Mplus (v7.0) was used to perform the statistical analyses, given its robust capabilities for handling structural equation models (SEM), CLPM, and latent variables in longitudinal data. Besides, multiple-group analyses were employed to assess for gender differences within the model. To determine if gender differences existed, each path was constrained, and the chi-square difference (Δχ^2^). A significant Δχ^2^ suggests the presence of gender differences in the model. Additionally, we examined other fit indices, including the change in the Comparative Fit Index (ΔCFI) and the Root Mean Square Error of Approximation (ΔRMSEA), as the chi-square statistic is sensitive to sample size. This allowed us to identify significant gender differences more precisely. Each path was then unconstrained to determine which exhibited gender differences.

Furthermore, we conducted measurement invariance tests to ensure that study constructs were measured equivalently across genders. We tested configural, metric, and scalar invariance and evaluated model fit through ΔCFI, ΔRMSEA, and Δχ^2^ to confirm invariance across groups.

Since our data was collected from university administrators in Putian, China, at two-time points, no complex sampling design was required. However, we rigorously screened the data for missing values and conducted attrition analysis to examine potential bias among non-responders across the two waves of data collection. Additionally, common-method bias was addressed by applying Harman’s single-factor test and confirming that no single factor accounted for more than 40% of the model’s variance. We also assessed multicollinearity using tolerance and VIF, ensuring it was not a concern in the analysis.

## Results

Table 1 presents descriptive estimates and findings from the reliability and validity analyses, including Cronbach’s alpha, composite reliability, and average variance extracted (AVE) for each study construct, evaluated at two distinct time points. The reliability metrics, specifically Cronbach’s alpha, exceed the 0.80 threshold for all constructs, demonstrating high internal consistency across both time points. The composite reliability values are also elevated, indicating that the constructs are highly reliable. The AVE values ≥0.50, indicating strong convergent validity. These results indicate that all constructs demonstrated strong reliability and convergent validity at both time points. The consistently high alpha, CR, and AVE values confirm that emotional intelligence, psychological empowerment, and work engagement were measured with precision and stability across the two waves.

**Table 1:** Reliability and validity analysis.

Study constructs	Cronbach’s alpha	Composite reliability	Average variance extracted
Emotional intelligence at T1	0.91	0.932	0.687
Psychological empowerment at T1	0.89	0.845	0.562
Work engagement at T1	0.89	0.872	0.598
Emotional intelligence at T2	0.90	0.897	0.665
Psychological empowerment at T2	0.89	0.855	0.629
Work engagement at T2	0.90	0.873	0.643

The results presented in Table 2 provide a thorough assessment of measurement invariance across four increasingly restrictive models: configural, metric, scalar, and residual invariance. The results supported full invariance across all tested levels. The configural model ( $\chi^{2}$ */df* = 1.52, RMSEA = 0.045, CFI = 0.951) was the baseline and demonstrated acceptable model fit, indicating that the basic factor structure was consistent across groups or time points. The metric invariance model, which constrains factor loadings to equality, yielded a slightly improved fit ( $\chi^{2}$ */df* = 1.38, RMSEA = 0.046, CFI = 0.952). The minimal changes in fit indices (∆RMSEA = 0.001, ∆CFI = 0.001) were well within recommended thresholds (∆CFI ≤ 0.01 and ∆RMSEA ≤ 0.015), supporting invariance of factor loadings. Similarly, scalar invariance, which constrains both factor loadings and item intercepts, also exhibited strong fit ( $\chi^{2}$ */df* = 1.33, RMSEA = 0.047, CFI = 0.953) with negligible change from the metric model (∆RMSEA = 0.001, ∆CFI = 0.001), suggesting that observed scores were comparable across conditions. Finally, residual invariance, the most stringent form, which additionally constrains item residuals, showed excellent model fit ( $\chi^{2}$ */df* = 1.08, RMSEA = 0.049, CFI = 0.952) and minimal degradation (∆RMSEA = 0.002, ∆CFI = −0.001). The minimal changes in RMSEA and CFI across increasingly restricted models confirm that the measurement structure remained equivalent over time, allowing valid comparison of the constructs across T1 and T2.

**Table 2:** Measurement invariance.

Model	$\chi^{2}$ /df	RMSEA	CFI	∆RMSEA	∆CFI
Configural invariance	1.52	0.045	0.951		
Metric invariance	1.38	0.046	0.952	0.001	0.001
Scalar invariance	1.33	0.047	0.953	0.001	0.001
Residual invariance	1.08	0.049	0.952	0.002	−0.001

Table 3 presents the bivariate correlations among emotional intelligence, psychological empowerment, and work engagement across two time points, revealing consistent and theoretically coherent patterns. At Time 1 (T1), emotional intelligence was significantly correlated with both psychological empowerment (*r* = 0.42, *p* < 0.001) and work engagement (*r* = 0.38, *p* < 0.001). In contrast, psychological empowerment showed the strongest association with work engagement (*r* = 0.47, p < 0.001), underscoring the immediate relevance of psychological empowerment for engagement. These associations remained statistically significant at Time 2 (T2), with emotional intelligence correlating with psychological empowerment (*r* = 0.44, *p* < 0.001) and work engagement (*r* = 0.39, *p* < 0.001), and psychological empowerment again showing a robust link with work engagement (*r* = 0.42, *p* < 0.001). Cross-lagged correlations from T1 to T2 further revealed that initial levels of emotional intelligence predicted subsequent psychological empowerment (*r* = 0.30, *p* < 0.01) and work engagement (*r* = 0.26, *p* < 0.01). In contrast, psychological empowerment at T1 significantly predicted work engagement at T2 (*r* = 0.33, *p* < 0.001), indicating modest but meaningful temporal influences. Additionally, all constructs demonstrated moderate stability over time, as reflected in significant correlations between corresponding T1 and T2 variables (e.g., work engagement T1–T2: *r* = 0.44, *p* < 0.001).

**Table 3:** Bivariate correlational analysis among study constructs at two different time points (T1-T2).

	EI (T1)	PE (T1)	WE (T1)	EI (T2)	PE (T2)	WE (T2)
EI (T1)	1					
PE (T1)	0.42***	1				
WE (T1)	0.38***	0.47***	1			
EI (T2)	0.35***	0.33***	0.29**	1		
PE (T2)	0.30**	0.41***	0.36***	0.44***	1	
WE (T2)	0.26**	0.33***	0.44***	0.39***	0.42***	1

***p < 0.001. **p < 0.01. EI, emotional intelligence; PE, psychological empowerment; WE, work engagement; T1, baseline period; T2, follow-up period.

The correlations showed that psychological empowerment had the strongest immediate association with work engagement at both time points. Emotional intelligence and psychological empowerment also showed moderate stability over time and meaningful predictive relationships across waves, supporting the reciprocal model tested in later analyses.

**Figure 1:**
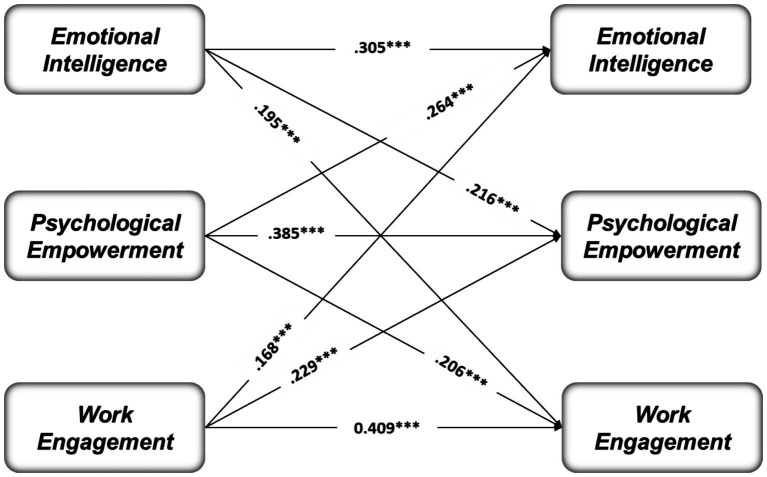
The cross-lagged path model analysis among study constructs at two different time points (T1–T2). For clarity and brevity in presentation, the coefficients of the error terms and correlation coefficients have been excluded.

Figure 1 shows the results of the overall cross-lagged path model among the study constructs at two different time points (T1-T2) over five-month intervals. At T1, emotional intelligence was found to predict psychological empowerment significantly at T2. The positive coefficient for this path indicates that individuals with higher levels of emotional intelligence at the first-time point tend to develop greater psychological empowerment over time. Similarly, emotional intelligence at T1 also predicted work engagement at T2, suggesting that those with higher emotional intelligence are more likely to experience increased work engagement later.

Conversely, psychological empowerment at T1 significantly predicted emotional intelligence at T2, indicating a reciprocal relationship between these variables. This reciprocal effect suggests that psychological empowerment at work contributes to the development of emotional intelligence over time. Furthermore, psychological empowerment at T1 also predicted work engagement at T2, showing that feeling empowered in one’s role at work leads to higher levels of work engagement later. The stability paths between T1 and T2 for all three constructs were significant, indicating that emotional intelligence, psychological empowerment, and work engagement are relatively stable across time. The coefficients suggest that these constructs maintain consistency, but the cross-lagged paths highlight that changes in one construct can influence the other over time.

Gender-based analyses indicated that the overall stability and reciprocal effects among emotional intelligence, psychological empowerment, and work engagement were largely similar for males and females (Table 4). All three constructs demonstrated moderate stability across the two waves for both groups. Although the stability of emotional intelligence was slightly higher among females, this was the only path showing a statistically significant difference; all other paths showed minimal numerical variation and did not differ significantly across gender.

**Table 4:** Gender difference analysis.

	Males	Females	Gender differences – Model comparisons		
Stability across T1-T2					
Emotional intelligence → Emotional intelligence	0.29 (0.02)	0.31 (0.02)	$\Delta \chi^{2}(1)=9.34,$ *p* < 0.001	Δ RMSEA = 0.001	ΔCFI =–0.001
Psychological empowerment → Psychological empowerment	0.37 (0.01)	0.38 (0.01)			
Work engagement → Work engagement	0.39 (0.01)	0.41 (0.01)			
Reciprocal effects across (T1-T2)					
Emotional intelligence → Psychological empowerment	0.21 (0.02)	0.18 (0.02)	$\Delta \chi^{2}(1)=4.01,$ *p* < 0.001	Δ RMSEA = 0.001	ΔCFI =0.000
Emotional intelligence → Work engagement	0.19 (0.02)	0.21 (0.02)			
Psychological empowerment → Emotional intelligence	0.26 (0.01)	0.24 (0.01)			
Psychological empowerment → Work engagement	0.20 (0.02)	0.21 (0.01)			
Work Engagement → Emotional Intelligence	0.16 (0.02)	0.15 (0.02)			
Work engagement → Psychological empowerment	0.20 (0.01)	0.22 (0.01)			

Results are based on multiple group analyses in which the overall model was initially tested for gender differences ( $\Delta \chi^{2}(9)=26.78,p<0.001)$ .

For the cross-lagged effects, emotional intelligence predicted later psychological empowerment and work engagement in both groups with very similar coefficients, and psychological empowerment showed comparable cross-lagged effects on emotional intelligence and work engagement. Reciprocal paths involving work engagement were also nearly identical across genders. Fisher’s r-to-z tests further confirmed that none of the corresponding correlations differed significantly between males and females (all *p* > 0.05).

Taken together, these findings indicate that the temporal and reciprocal relationships among the three constructs operate similarly across genders, with only a small difference observed in the stability of emotional intelligence. Overall model-fit indices (ΔRMSEA, ΔCFI) support equivalence across gender groups.

Table 4 summarizes the gender-specific path coefficients for the longitudinal model. Across both groups, emotional intelligence, psychological empowerment, and work engagement showed significant stability from T1 to T2. Most cross-lagged paths were also significant and displayed comparable magnitudes across genders. Although females showed slightly stronger effects in a few paths (e.g., EI → WE), the model-comparison tests indicated that none of the differences were statistically significant. Overall, the pattern of associations was largely consistent for males and females.

## Discussion

The present study aimed to address key gaps in the literature by exploring the reciprocal relationships between emotional intelligence, psychological empowerment, and work engagement among university administrators over time. While previous studies have examined these constructs individually, few have examined their dynamic interactions longitudinally, particularly within a university administration setting. This study fills that gap by employing a CLPM design, which allows us to examine the stability of these variables over time and their reciprocal effects across two-time points. Furthermore, the study contributes to the literature by assessing potential gender differences in these relationships, an aspect that is largely underexplored in this population.

The results revealed that emotional intelligence positively predicted both psychological empowerment and work engagement over time, while psychological empowerment also predicted changes in emotional intelligence and work engagement. The stability of emotional intelligence, psychological empowerment, and work engagement over time was significant, indicating that these traits and behaviors remain relatively stable but can also meaningfully influence one another. Although slight numerical differences were observed between males and females, the multi-group SEM comparison showed that these differences were not statistically significant. Overall, the stability and reciprocal relationships among emotional intelligence, psychological empowerment, and work engagement were highly similar across genders.

The longitudinal contribution of this study is significant, as it provides evidence of how emotional intelligence, psychological empowerment, and work engagement mutually reinforce one another over time. Much of the existing research has examined these constructs using cross-sectional designs, which capture associations at a single time point but cannot reflect their dynamic interplay. While earlier foundational work highlighted reciprocal relations between empowerment and engagement (Xanthopoulou et al., 2007), more recent longitudinal studies have similarly shown that personal and job resources interact over time to shape employee well-being and performance (Mazzetti et al., 2023; Kim et al., 2018; Ravichandran et al., 2011). The present findings extend this line of research by demonstrating that emotional intelligence also contributes to changes in psychological empowerment and engagement across time, underscoring its importance within administrative environments where complex interpersonal and organizational demands are routine.

The positive longitudinal association between emotional intelligence and work engagement observed in this study is consistent with the broader body of research highlighting emotional intelligence as a key personal resource for sustaining adaptive work functioning (Ravichandran et al., 2011; Extremera et al., 2018; George et al., 2022). For university administrators, who routinely manage demanding interpersonal situations involving staff, students, and institutional processes, the capacity to regulate emotions is particularly valuable for maintaining engagement (Schutte and Loi, 2014). Our findings extend prior work by demonstrating that emotional intelligence not only correlates with engagement at a single time point but also predicts increases in engagement over time. Although slight numerical differences emerged between males and females, these variations were not statistically significant, indicating that the role of emotional intelligence in fostering engagement operates similarly across genders in this context.

Similarly, the reciprocal relationship between psychological empowerment and emotional intelligence suggests that feeling empowered at work may enhance one’s development. This finding is particularly relevant for university administrators, who are often tasked with making autonomous decisions and leading initiatives that require both emotional and psychological resilience. Previous research has shown that psychological empowerment is linked to greater job satisfaction and performance (Ölçer and Florescu, 2015; Sun, 2016; Hechanova et al., 2006). Our study builds on this by demonstrating that empowerment can also promote emotional growth, which, in turn, fosters greater workplace engagement.

Although small numerical differences appeared in the stability of emotional intelligence and its reciprocal association with work engagement, these variations were not statistically significant in the multi-group analyses. Therefore, any gender-related patterns should be interpreted cautiously. Prior literature does suggest that women in administrative and leadership roles often encounter higher emotional demands, which may lead them to rely more heavily on emotional intelligence as a coping resource (Sherman, 2022; Kassim et al., 2022). Goleman (1996) also noted that emotional intelligence can be an important predictor of leadership effectiveness among women. While these insights offer possible explanations for the slight numerical trends observed in our sample, the present findings do not provide empirical evidence of meaningful gender differences in these pathways.

Consistent with the statistical results, psychological empowerment and work engagement demonstrated highly similar effects across male and female administrators, with no significant gender-based differences. This aligns with prior research showing that psychological empowerment operates as a robust motivator across genders, supporting intrinsic motivation and job satisfaction equally for men and women (Kim et al., 2018; Mazzetti et al., 2023). In the context of university administration, where autonomy, competence, and impact are central to role performance, empowerment appears to function as a universal resource that contributes similarly to engagement regardless of gender. While the current model focused on the core personal and job resources central to our research aims, it is likely that additional psychological mechanisms, such as burnout reduction, increased job satisfaction, or supportive leadership, play important roles in shaping the observed relationships. Examining these pathways would allow future studies to capture the broader motivational processes proposed by the JD–R model.

### Strengths and limitations

This study has several limitations that should be acknowledged. First, although a two-wave longitudinal design was used, the six-month interval may not fully capture the temporal complexity of changes in emotional intelligence, psychological empowerment, and work engagement. Multi-wave designs with longer lags would allow for stronger causal inferences. Second, the sample was restricted to university administrators in Putian, which may limit the generalizability of the findings to other institutional or cultural contexts. Replication across diverse settings is recommended. Third, reliance on self-reported data raises the possibility of common-method and social-desirability bias, despite statistical evidence indicating no substantial method bias. Future studies may benefit from multi-source or objective measures. Fourth, although multidimensional scales were modeled using item parceling, examining specific dimensions of emotional intelligence and psychological empowerment may offer more fine-grained insights. Finally, the study did not incorporate additional demographic or JD–R variables, such as age, job satisfaction, burnout, or organizational support, that may function as mediators or moderators. These mechanisms should be explored in future multi-wave studies to provide a more comprehensive account of how personal and job resources shape work engagement.

Despite these limitations, the study offers several notable strengths. The longitudinal design and use of cross-lagged panel modeling provide valuable insight into the directionality and reciprocal nature of relationships among emotional intelligence, psychological empowerment, and work engagement, an area that has been understudied in administrative populations. The inclusion of a sizable sample of university administrators also contributes rare empirical evidence from a professional group that receives limited research attention. By modeling temporal dynamics rather than relying solely on cross-sectional data, the study strengthens theoretical understanding of how personal and job resources operate over time within the JD–R framework.

### Study implications

This study contributes to the literature by providing longitudinal evidence of reciprocal relationships among emotional intelligence, psychological empowerment, and work engagement. By demonstrating that these resources influence one another over time, the findings support dynamic, rather than static, interpretations of psychological processes within the JD–R framework. The results also highlight the importance of modeling temporal pathways to understand how personal and job resources co-develop in administrative settings.

The findings have clear relevance for university management and human resource development. Because emotional intelligence and psychological empowerment both predicted increases in work engagement over time, institutions should prioritize interventions that strengthen these psychological resources. Emotional intelligence can be enhanced through structured training, coaching, or professional development workshops focused on emotion regulation, communication, and interpersonal problem-solving. Similarly, psychological empowerment can be supported by increasing autonomy, providing meaningful responsibilities, and recognizing employees’ contributions. These actions can help administrators remain engaged despite demanding workloads and emotionally complex roles. Additionally, because the temporal patterns were broadly similar across genders, institutions do not need highly differentiated gender-specific interventions. Instead, broad organizational strategies that enhance emotional intelligence and empowerment are likely to benefit the entire administrative workforce. Investing in these resources can support administrators’ well-being, reduce turnover intentions, and promote sustained performance in university operations.

## Conclusion

In conclusion, this study demonstrates that emotional intelligence, psychological empowerment, and work engagement mutually reinforce one another over time among university administrators. Emotional intelligence emerged as an important personal resource that contributes to increases in both psychological empowerment and engagement across the semester. Although small numerical differences were observed across genders, these differences were not statistically significant, suggesting that the observed relationships operate similarly for male and female administrators. Overall, the findings highlight the value of fostering emotional and psychological resources in administrative work environments to promote sustained engagement and well-being. Future research using multi-wave longitudinal designs and more diverse institutional contexts is encouraged to deepen understanding of these dynamic processes.

## Data Availability

The raw data supporting the conclusions of this article will be made available by the authors, without undue reservation.
